# Out-of-hospital cardiac arrest in children: an epidemiological study based on the German Resuscitation Registry identifying modifiable factors for return of spontaneous circulation

**DOI:** 10.1186/s13054-023-04630-3

**Published:** 2023-09-07

**Authors:** Stephan Katzenschlager, Inga K. Kelpanides, Patrick Ristau, Matthias Huck, Stephan Seewald, Sebastian Brenner, Florian Hoffmann, Jan Wnent, Jo Kramer-Johansen, Ingvild B. M. Tjelmeland, Markus A. Weigand, Jan-Thorsten Gräsner, Erik Popp

**Affiliations:** 1 Department of Anaesthesiology, Heidelberg University Hospital, Im Neuenheimer Feld 420, 69120 Heidelberg, Germany; 2 Department of Research and Development, Division of Emergencies and Critical Care, Oslo University Hospital, Oslo, Norway; 3 Faculty of Medicine, Institute of Clinical Medicine, University of Oslo, Oslo, Norway; 4 Institute for Emergency Medicine, University Hospital Schleswig-Holstein, Kiel, Germany; 5 Department of Anesthesiology and Intensive Care Medicine, University Hospital Schleswig-Holstein, Campus Kiel, Kiel, Germany; 6 Department of Pediatric and Adolescent Medicine, University Clinic Carl Gustav Carus, Dresden, Germany; 7 Paediatric Intensive Care and Emergency Medicine, Dr. Von Hauner Children’s Hospital, Ludwig-Maximilians-University, Munich, Germany; 8 School of Medicine, University of Namibia, Windhoek, Namibia; 9 Division of Prehospital Services, Oslo University Hospital, Oslo, Norway

**Keywords:** Out-of-hospital cardiac arrest, Resuscitation, Paediatric cardiac arrest, Emergency medical service, Epidemiology

## Abstract

**Aim:**

This work provides an epidemiological overview of out-of-hospital cardiac arrest (OHCA) in children in Germany between 2007 and 2021. We wanted to identify modifiable factors associated with survival.

**Methods:**

Data from the German Resuscitation Registry (GRR) were used, and we included patients registered between 1st January 2007 and 31st December 2021. We included children aged between > 7 days and 17 years, where cardiopulmonary resuscitation (CPR) was started, and treatment was continued by emergency medical services (EMS). Incidences and descriptive analyses are presented for the overall cohort and each age group. Multivariate binary logistic regression was performed on the whole cohort to determine the influence of (1) CPR with/without ventilation started by bystander, (2) OHCA witnessed status and (3) night-time on the outcome hospital admission with return of spontaneous circulation (ROSC).

**Results:**

OHCA in children aged < 1 year had the highest incidence of the same age group, with 23.42 per 100 000. Overall, hypoxia was the leading presumed cause of OHCA, whereas trauma and drowning accounted for a high proportion in children aged > 1 year. Bystander-witnessed OHCA and bystander CPR rate were highest in children aged 1–4 years, with 43.9% and 62.3%, respectively. In reference to EMS-started CPR, bystander CPR with ventilation were associated with an increased odds ratio for ROSC at hospital admission after adjusting for age, sex, year of OHCA and location of OHCA.

**Conclusion:**

This study provides an epidemiological overview of OHCA in children in Germany and identifies bystander CPR with ventilation as one primary factor for survival.

*Trial registrations* German Clinical Trial Register: DRKS00030989, December 28th 2022.

**Graphical Abstract:**

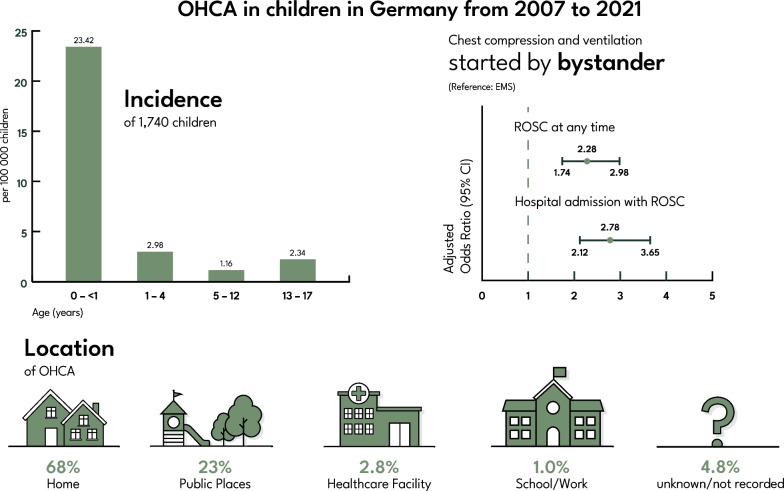

**Supplementary Information:**

The online version contains supplementary material available at 10.1186/s13054-023-04630-3.

## Introduction

Out-of-hospital cardiac arrest (OHCA) in children is a rare event with a high burden for the victim, caregivers, rescue personnel and society. Globally, the incidence of OHCA in children remains low, with around 8/100,000 inhabitants per year [1–3]; however, incidence in < 1-year-olds is substantially higher [3–5]. The low incidence compared to OHCA in adults, combined with ethical issues, has resulted in a lack of interventional studies on OHCA in children [6]. Survival rates have increased for in-hospital cardiac arrests, but this success did not show higher rates of survival with good neurologic outcome [7]. Emergency medical service (EMS) crews are challenged by the heterogeneity of different age groups, facing wide anatomical and physiological ranges. Aetiologies of OHCA in children differ from those in adults, as hypoxia is one of the leading presumed causes [8, 9]. However, the aetiology varies across the paediatric OHCA population, with an increase in trauma in the adolescent group and a decrease in respiratory failure and hypoxemia. Due to those differences, compared to adults, treatment changes and the focus lie more on ventilation and oxygenation rather than early defibrillation [10]. As opposed to the assumption of a cardiac cause in adult OHCA without any other obvious causes, a cause which would predominate in the child has not been identified, leaving caregivers and researchers with another challenge in the endeavour to improve the treatment of OHCA in children.

Although an Utstein recommendation on reporting OHCA in children exists [11], comparability between different studies is limited due to the different age groups reported. It is crucial to differentiate between respiratory and cardiopulmonary arrest; however, this is not always possible, especially in retrospective cohort studies. While there is an increase in the analyses of specific treatment strategies [2, 12, 13], recent large epidemiological studies across a whole nation or a continent are lacking. These can help EMS systems to tailor their paediatric advanced life support (PALS) training and provide the best possible care.

The aim of this study is to give an epidemiological overview of OHCA in children across Germany and identify factors that are associated with hospital admission with return of spontaneous circulation (ROSC).

## Methods

### Study design

In this retrospective cross-sectional cohort study, patients under the age of 18 with an OHCA from 1 January 2007 to 31 December 2021 were included. This analysis was performed with data from GRR and followed the Strengthening the Reporting of Observational studies in Epidemiology (STROBE) guidelines [14].

### German Resuscitation Registry (GRR)

GRR covered approximately 37.4% (31.1 million) of the population of Germany by the end of 2021 and is one of Europe biggest cardiac arrest registries [15, 16], significantly contributing to the scarce population data and literature on OHCA in children.

Out of the proportion covered within GRR, 16.7% (5.2 million) were under the age of 18 years. This increased from only 1.3 million children covered within the registry in 2007 (Additional file 1: Table S1a). To date, about 100 EMS organisations are participating in GRR on a voluntarily basis. In addition to sites in Germany, some Austrian and Swiss EMS systems choose to participate in the GRR. For the acquisition of pre-hospital data, a dataset with 118 variables in concordance with the Utstein recommendations exists [17]. This dataset focuses on time intervals, pre-hospital interventions, patient specific factors such as age, known diseases, pre-emergency state according to the American Society of Anesthesiology physical status classification (ASA classification) [18] and patient status at hospital admission. ASA classification ranged from 1 to 5 and was determined by the treating physician according to the information available on scene. Pre-existing conditions were entered into GRR by the treating physician on scene. Due to the structure of the database, up to three existing conditions could be entered. ‘Reference centres’ are EMS systems that provide high data quality for both pre- and in-hospital data [19]. All pre-hospital data are collected and entered into GRR by the treating emergency physician.

### EMS system in Germany

The EMS system is a two-tier system with ambulance crews consisting of at least a paramedic (German: 'Notfallsanitäter') and an emergency medical technician (EMT) (German: 'Rettungssanitäter'). The second tier that is always dispatched to suspected cardiac arrests is a physician response unit staffed with an emergency physician and a paramedic. The physician response unit can either be a rapid response car or an air ambulance. In addition, some regions have implemented first responder systems. Paramedics are trained in advanced life support (ALS) treatment of OHCA in adults and paediatrics. Ultimately, the emergency physician makes the treatment decisions on scene.

### Inclusion and exclusion criteria

We included all patients between > 7 days and 17 years of age suffering an OHCA with resuscitation attempts and continued treatment by EMS in Germany. No restrictions for sex or the aetiology of OHCA were applied.

Data from participants outside of Germany and data on patients with an incorrect or missing age were excluded. Furthermore, cases where the patient was declared deceased on arrival (DoA) by the EMS team without resuscitation attempts by a bystander or EMS were excluded. Descriptive baseline characteristics of patients <  = 7 days and children declared DoA are presented in the supplementary material (Additional file 1: Tables S2 and S3).

### EMS time intervals

The following time intervals were calculated based on the times reported by the EMS system:

- Response time, defined as the interval from the emergency call started until the first EMS team arrived at the destination defined by dispatch.
- On-scene time, defined as the interval from the first EMS team on scene until transport to the hospital was either started or the patient was declared dead.
- Transport time, defined as the interval from the start of the transport by EMS until arrival at the hospital.
- EMS treatment time, defined as the interval from the arrival of the first EMS team until arrival at the hospital (= on-scene interval + transport interval). When the patient diseased and was not transported, EMS treatment duration was defined as the on-scene interval.
- Duration to first shock, defined as the interval from the emergency call until anyone delivered the first shock in patients with shockable rhythm.

### Data synthesis and statistical analysis

All analyses were performed using SPSS (Statistical Product and Services Solutions, Version 28, SPSS Inc., Chicago, IL, USA). Baseline and demographic characteristics are presented as incidence per 100,000 children and frequencies for the overall cohort and as frequencies only for the age-specific subgroups. Continuous variables are summarised as means with standard deviations (SD) or medians and interquartile ranges (IQR).

Incidence was calculated based on the official population calculations from the German Federal Statistical Office [20]. We assumed that the proportion of children is equally distributed across Germany. The complete data for incidence calculation is available in the supplementary material (Additional file 1: Table S1b). Yearly incidence was calculated by using the formula $\frac{paediatric\,\, OHCA\,\, per\,\, age \,\,group\,\, (n)}{per\,\, age\,\, group\,\, within\,\, registry\,\, (n)\,\, per\,\, \mathrm{100,000}}$ (e.g. Additional file 1: Table S1a, b for 2007 and age group < 1 year: 11/0.69). Overall incidences are reported as the mean across the study period. Reporting of incidences were chosen for better comparisons with future studies of different registries, cohorts and EMS systems in accordance with recent recommendations [21, 22].

The four age groups were categorised according to the latest Utstein recommendation, with > 7 days to < 1 year (henceforth named < 1 year), 1 to 4 years, 5 to 12 years and 13 to 17 years [11]. Treatment before EMS arrival was defined as interventions performed either by bystanders or first responders. Night-time was defined between 10 pm to 6am. First rhythm assessed was defined as the first rhythm when CPR was commenced. Bradycardia was defined as a heartrate below 60 beats per minute; due to the structure of GRR, this is only possible in patients below 14 years of age.

EMS time intervals were analysed using Mann–Whitney-U due to the non-normal distribution of the data. The effect size was calculated as the differences between pseudomedians with nonparametric 95% confidence intervals (CI) using the Hodges–Lehmann method [23].

Multivariate binary regression analysis was used to calculate the adjusted odds ratio (aOR) for the factors 'chest compression started by', 'chest compression and ventilation started by', 'OHCA witnessed by' and 'night-time' for the outcome 'ROSC at hospital admission'. Regression analysis was adjusted for age (per year, continuous variable), sex, year of OHCA (continuous variable) and location of OHCA.

In GRR, parameters from the Utstein core dataset are mandatory, while the remainder is voluntary, thus leading to the decision to not impute missing data. A two-sided level of significance < 0.05 was considered statistically significant.

### Steering committee and ethical approval

According to the regulations of the GRR, a study protocol was drafted and approved by the scientific advisory board before the beginning of the data analysis (Ref. number: 2022–06).

This study was approved by the University Heidelberg ethics committee with reference number S-085/2022 and registered at the German Clinical Trial Register (www.drks.de—study number DRKS00030989).

## Results

### Demographic overview

**Fig. 1:**
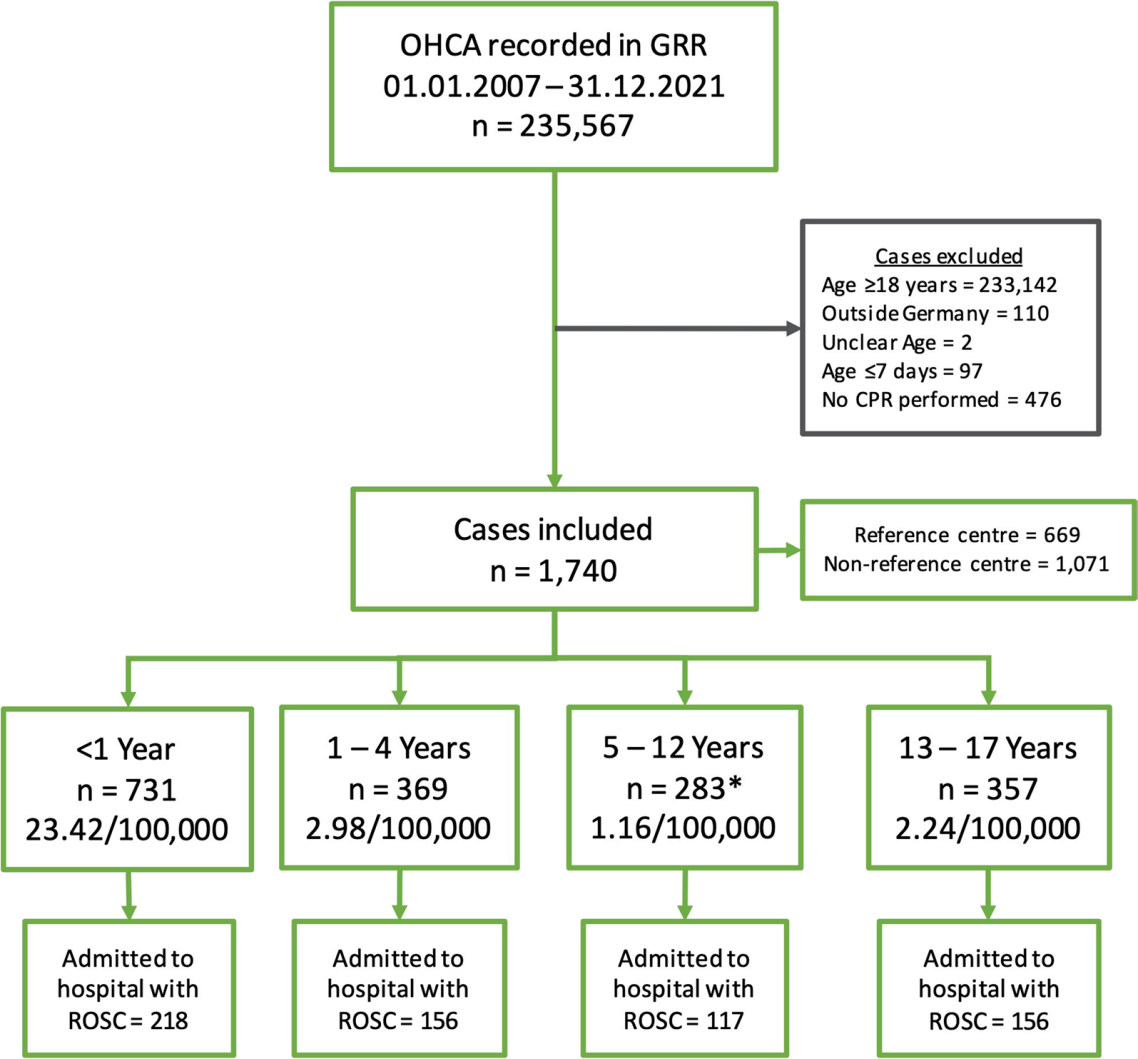
Study flowchart demonstrating included cases and incidences per age group; *1 case with an unknown immediate outcome. OHCA = out-of-hospital cardiac arrest; GRR = German Resuscitation Registry; CPR = cardiopulmonary resuscitation; ROSC = return of spontaneous circulation

Over the 15 years, 235,537 OHCAs were recorded in GRR. The complete list of excluded cases with reasons is available in Fig. 1.

**Fig. 2:**
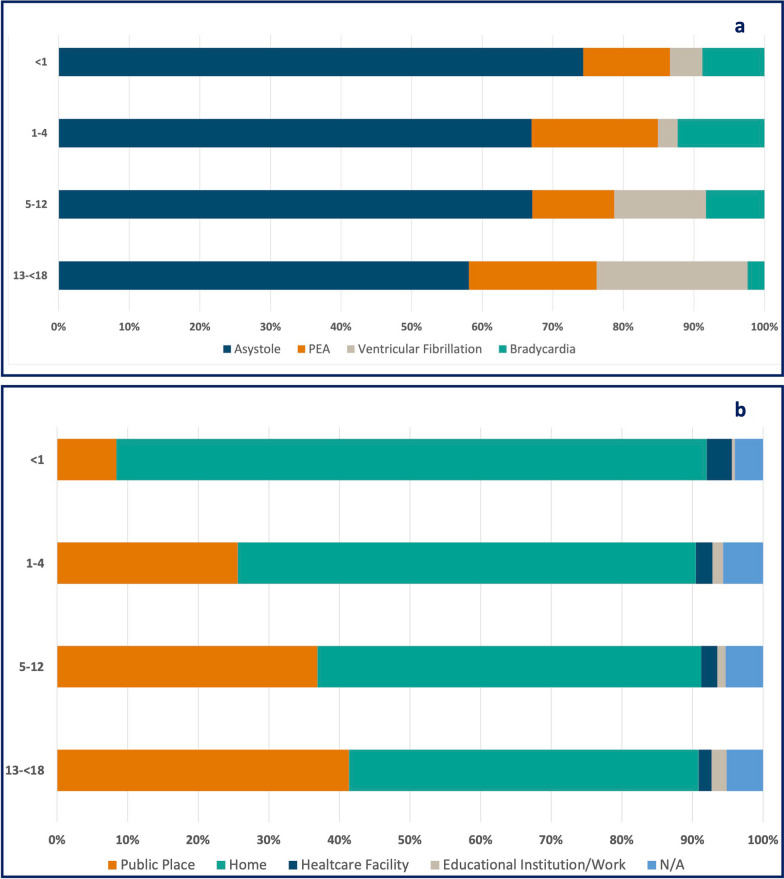
**a** Proportion of the first rhythm assessed by age group, **b** location of arrest according to age group; PEA = pulseless electric activity, N/A = not available or missing

The main reason for exclusion was age ≥ 18 years (n = 233,142; 98.9%). Furthermore, 476 cases where no CPR was performed due to obvious signs of death and 97 cases with age ≤ 7 days were excluded. (Additional file 1: Tables S2 and S3). In total, 1,740 datasets of children suffering from OHCA and receiving CPR were included in this study. This results in an overall incidence across all age groups of 3.08/100,000 children. There was slight variance within the incidence per year with a minimum of 1.68 in 2010 and a maximum of 4.26 in 2019. Incidence was highest in children aged < 1 year, with 23.42/100,000 (Fig. 1). Non-shockable rhythms had the highest overall incidence, with 2.46/100,000 for initial rhythm. In contrast, the incidence of ventricular fibrillation (VF) was 0.29/100,000. The highest proportion of VF as initial rhythm was found in children aged 13 to 17 years, with 21.4% (n = 74) (Fig. 2a, Table 1).

**Table 1:** Baseline characteristics

Characteristic Incidence/100,000	Overall N = 1,740 3.08/100,000	< 1 Year n = 731 23.42/100,000	1–4 Years n = 369 2.98/100,000	5–12 Years n = 283 1.16/100,000	13–17 Years n = 357 2.24/100,000
Age, median (IQR)	1.8 years (0.3 to 10.9)	93 days (40 to 171)	2.2 years (1.5 to 3.2)	8.7 years (6.6 to 11.1)	16.2 years (14.9 to 17.1)
Sex, n female (%)	686 (39.4%)	288 (39.4%)	160 (43.4%)	107 (37.8%)	131 (36.7%)
*Location of OHCA, n (%)/Incidence*^#^					
Healthcare Facility outside hospital	45 (3%)/0.07	25 (4%)	8 (2%)	6 (2%)	6 (2%)
Public place	378 (23.2%)/0.66	59 (8%)	86 (25.6%)	97 (36.9%)	136 (41.3%)
Home	1111 (68.2%)/1.91	587 (83.6%)	218 (64.9%)	143 (54.4%)	163 (49.5%)
Educational Institution/Workplace	18 (1%)/0.03	3 (0.4%)	5 (2%)	3 (1%)	7 (2%)
Not recorded	78 (5%)/0.23	28 (4%)	19 (6%)	14 (5%)	17 (5%)
Presumed cause, n (%)/Incidence					
Cardiac	241 (13.9%)/0.43	20 (3%)	29 (8%)	43 (15.2%)	63 (17.6%)
Trauma	193 (11.1%)/0.37	22 (3%)	38 (10.3%)	45 (15.9%)	88 (24.6%)
Drowning	134 (8%)/0.22	7 (1%)	72 (19.5%)	46 (16.2%)	9 (3%)
Hypoxia	540 (31.0%)/0.91	229 (31.3%)	143 (38.7%)	83 (29.3%)	85 (23.8%)
Intoxication	24 (1%)/0.04	3 (0.4%)	2 (0.5%)	1 (0.4%)	18 (5%)
SIDS	211 (12.1%)/0.38	202 (27.6%)	9 (2%)*	-	-
Other	156 (9%)/0.31	51 (7%)	37 (10.0%)	34 (12.0%)	34 (9%)
Unknown	241 (13.9%)/0.43	111 (15.2%)	39 (10.5%)	31 (11.0%)	60 (16.8%)
Relation to sports activity, n (%)	40 (2%)	5 (1%)	1 (0.3%)	8 (3%)	26 (7%)
Night-time (10 pm to 6am), n (%)^##^	369 (21.2%)	166 (23.3%)	63 (17.7%)	55 (20.1%)	85 (24.4%)
Witnessed by, n (%)/Incidence					
Bystander	632 (36.3%)/1.07	214 (29.3%)	162 (43.9%)	109 (38.5%)	147 (41.2%)
First responder	22 (1%)/0.04	11 (2%)	5 (1%)	3 (1%)	3 (1%)
EMS	113 (7%)/0.19	46 (6%)	16 (4%)	22 (8%)	29 (8%)
Unwitnessed	973 (55.9%)/1.80	460 (62.9%)	186 (50.4%)	149 (52.7%)	178 (49.9%)
Bystander CPR, n (%)	911 (56%)	368 (54%)	230 (65.1%)	149 (57.1%)	164 (50.0%)
Telephone assisted CPR, n (%)	444 (25.5%)	210 (28.7%)	109 (29.5%)	55 (19.4%)	70 (19.6%)
First assessed rhythm, n (%)/Incidence^###^					
Asystole	1155 (68.3%)/2.05	529 (74.3%)	240 (67.0%)	186 (67.1%)	200 (58.1%)
PEA	245 (14.5%)/0.41	87 (12.3%)	64 (17.9%)	32 (11.6%)	62 (18.1%)
Ventricular fibrillation	153 (9%)/0.29	33 (5%)	10 (3%)	36 (13.0%)	74 (21.4%)
Bradycardia	138 (8%)/0.23	63 (9%)	44 (12.3%)	23 (8%)	8 (2%)
Pre-emergency status, n (%)/Incidence					
ASA I	690 (39.7%)/1.02	304 (41.6%)	145 (39.3%)	102 (36.0%)	139 (38.9%)
ASA II	145 (8%)/0.21	53 (7%)	27 (7%)	21 (7%)	44 (12.3%)
ASA III	209 (12.0%)/0.31	97 (13.3%)	49 (13.3%)	39 (13.8%)	24 (7%)
ASA IV	135 (8%)/0.19	37 (5%)	33 (9%)	36 (12.7%)	29 (8%)
ASA V	3 (0.2%)/0.00	1 (0.1%)	1 (0.3%)	-	1 (0.3%)
Not recorded	558 (32.1%)	239 (32.7%)	114 (30.9%)	85 (30.0%)	120 (33.6%)
Pre-existing conditions, n (%)/ Incidence					
Cardiac	181 (10.4%)/0.27	99 (13.5%)	34 (9%)	29 (10.2%)	19 (5%)
Pulmonary	120 (7%)/0.20	56 (8%)	29 (8%)	25 (9%)	18 (5%)
Metabolic	80 (5%)/0.12	35 (5%)	16 (4%)	14 (5%)	15 (4%)
Malignancy	19 (1%)/0.03	10 (1%)	4 (1%)	3 (1%)	2 (1%)
Neurologic	204 (11.7%)/0.33	46 (6%)	56 (15.2%)	54 (19.1%)	48 (13.4%)
Immunodeficiency	21 (1%)/0.03	5 (1%)	6 (2%)	6 (2%)	4 (1%)

Percentages were rounded, wherefore they might not add up to 100%. # Location of OHCA: 110 cases are missing. Respective case numbers are: < 1 Year: 702, 1–4 Years: 336, 5–12 Years: 263, 13–17 Years: 329. ## Night-time: 51 cases are missing. Respective case numbers are: 0- < 1 Year: 712, 1–4 Years: 356, 5–12 Years 273, 13–17 Years: 348. ### First rhythm assessed: 49 cases are missing. Respective case numbers are: 0- < 1 Year: 712, 1–4 Years: 358, 5–12 Years: 277, 13–17 Years: 344. *SIDS was only recorded in children up to the age of 2 years. *OHCA* out-of-hospital cardiac arrest; *SIDS* sudden infant death syndrome; *EMS* emergency medical service; *CPR* cardiopulmonary resuscitation; *ASA* American Society of Anaesthesiologists Classification

Across all age groups, ‘home’ was the most common location of OHCA with 1.91/100,000. In the age group 13 to 17 years, ‘home’ and ‘public place’ had proportions of 49.5% (n = 163) and 41.3% (n = 136), respectively (Fig. 2b). The most common presumed cause of OHCA was hypoxia and cardiac with the overall incidences of 0.91 and 0.43/100,000, respectively. Presumed cause of OHCA differed between the age groups. For children < 1 year, hypoxia and sudden infant death syndrome (SIDS) were the leading presumed causes of OHCA. In older children, trauma, drowning and cardiac causes are increasing (Table 1).

Across the entire time span, OHCAs were mainly unwitnessed (1.80/100,000); however, this declined from 2007 (2.19) to 2021 (1.88). Bystander-witnessed OHCAs increased from an incidence of 0.51 in 2007 to 1.11 in 2021. This is equally distributed across all age groups. Hospital admission with ongoing CPR had higher incidences in more recent years (2020, 2021) with 0.70 and 0.75/100,00 compared to the first two years (2007, 2008) with 0.66 and 0.40, respectively.

The full demographic results including pre-existing conditions and pre-emergency status are presented in Table 1.

### Pre-hospital treatment

**Fig. 3:**
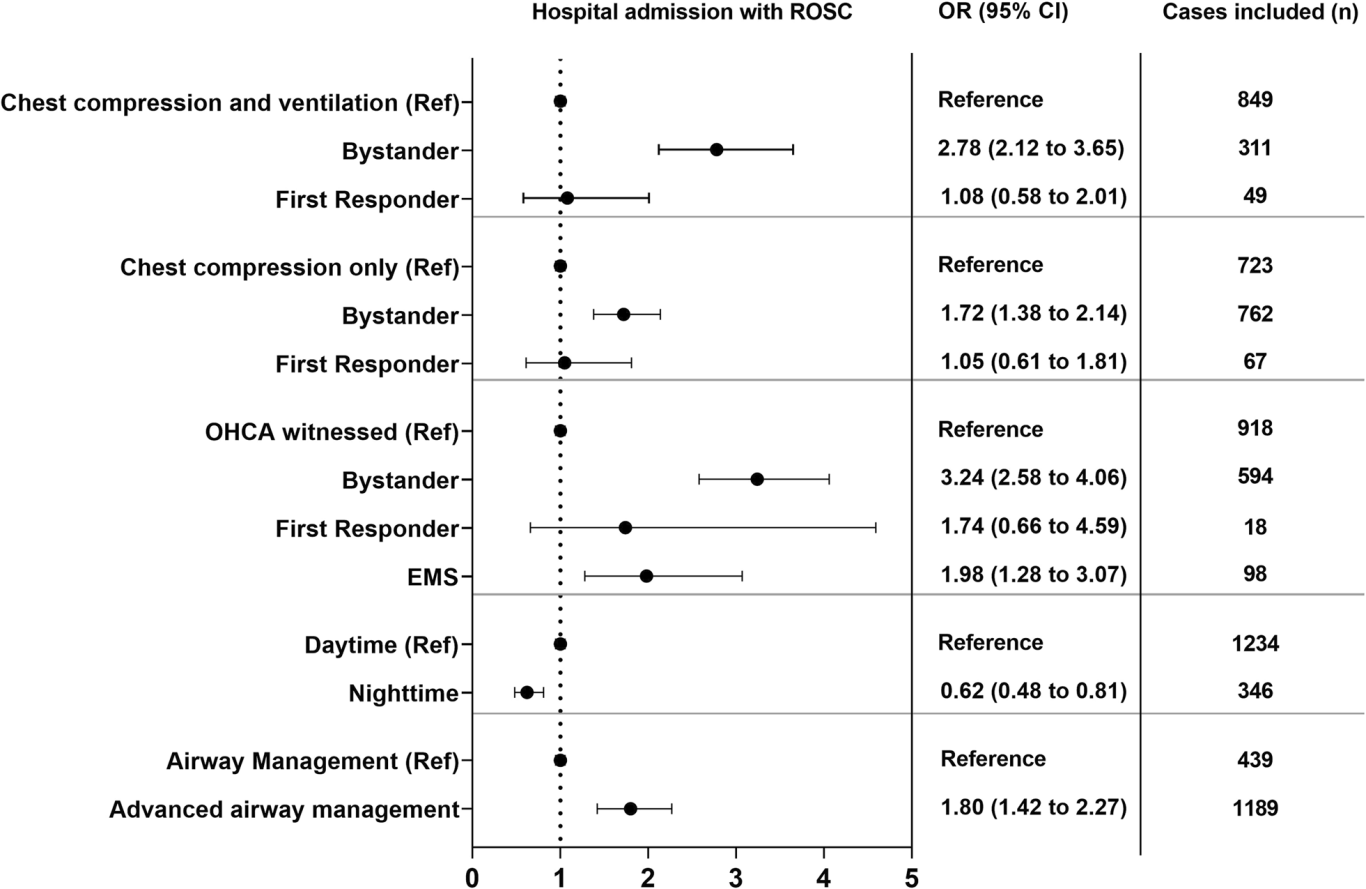
Forest plot with adjusted odds ratio for hospital admission with ROSC. Reference groups are: chest compression and ventilation: EMS; chest compression only: EMS; OHCA witnessed: unwitnessed; daytime: 06am to 10 pm; airway management: bag-mask ventilation; OHCA = out-of-hospital cardiac arrest; EMS = emergency medical service; ROSC = return of spontaneous circulation; OR = odds ratio; CI = confidence interval

Referring to the first link in the chainmail of survival [24], bystanders performed chest compression only (CCO) in 20.0% (n = 348) of all cases. Chest compression and ventilation (CCV) were performed in 18.6% (n = 324), with a higher proportion compared to CCO in 1 to 4 and 5 to 12 years (27.6% vs 20.1% and 19.1% vs 17.3%, respectively). Out of 235 performed defibrillations, EMS performed 97.5% (n = 229). In total, six defibrillations were performed before EMS arrival, four of those by first responders. Refractory VF, defined as > 3 shocks, was stated in 21.3% (n = 50) of all cases, with the largest proportion in the 13 to 17 years group (26.5%, n = 26). A similar proportion was found in the < 1 year age group with 23.4% (n = 15). Endotracheal intubation (ETI) was the most frequently used strategy for airway management in all age groups. In comparison, bag-mask ventilation during CPR only was used in 3.8% (n = 67) of all cases. Any type of advanced airway management during pre-hospital treatment achieved higher aOR for hospital admission with ROSC (Fig. 3). Regarding supraglottic airway devices, laryngeal tubes (LT) were used in 54.7% (175/320) (Additional file 1: Table S4c). Although an airway management device was used in most cases, end-tidal CO2 (etCO2) at hospital admission was not routinely reported, with an increase over the study period (Additional file 1: Table S1b). Administration of adrenaline using intraosseous (i.o.) access was highest in the youngest age group (< 1 year) with 42.1% and declined to 21.0% in the oldest age group (13 to 17 years). Full details on pre-hospital treatment are presented in Table 2 and Additional file 1: Table S5.

**Table 2:** Pre-hospital treatment

	Overall N = 1,740	< 1 Year n = 731	1–4 Years n = 369	5–12 Years n = 283	13–17 Years n = 357
*Treatment before EMS arrival, n (%)*					
Chest compression only	362 (20.8%)	145 (19.8%)	76 (20.6%)	54 (19.1%)	87 (24.4%)
Ventilation only	35 (2%)	11 (2%)	10 (3%)	3 (1%)	11 (3%)
Chest compression and ventilation	355 (20.4%)	150 (20.5%)	108 (29.3%)	60 (21.2%)	39 (10.9%)
Defibrillation*	6 (0.3%)	1 (0.1%)	–	2 (1%)	3 (1%)
*Airway management, n (%)*					
BMV only	67 (4%)	26 (4%)	8 (2%)	8 (3%)	25 (7%)
SGA**	320 (23%)	150 (26%)	59 (21%)	34 (15%)	77 (25%)
Endotracheal Intubation**	1070 (77%)	420 (74%)	221 (79%)	193 (85%)	236 (75%)
End-tidal CO2 at admission**	425 (31%)	119 (21%)	103 (37%)	85 (37%)	118 (38%)
*Medication*					
Adrenaline, N	1579	673	360	269	277
i.v. n (%)***	873 (55%)	344 (51%)	177 (49%)	156 (58%)	196 (71%)
i.o. n (%)***	668 (42%)	308 (46%)	173 (48%)	112 (42%)	75 (21%)
Atropine, N	176	52	56	37	31
i.v. n (%)***	106 (60%)	28 (54%)	31 (55%)	23 (62%)	24 (77%)
i.o. n (%)***	65 (37%)	23 (44%)	23 (41%)	14 (38%)	5 (16%)
Amiodarone, N	70	17	4	10	39
i.v. n (%)***	61 (87%)	17 (100%)	2 (50%)	7 (70%)	35 (90%)
i.o. n (%)***	9 (13%)	–	2 (50%)	3 (30%)	4 (10%)
Crystalloid Fluids, n (%)	964 (55.4%)	352 (48.2%)	194 (52.6%)	189 (66.8%)	229 (64.1%)
Thrombolysis, n (%)	19 (1%)	8 (1%)	–	–	10 (3%)
Defibrillation, total (%)	n = 235 (13.5%)	n = 64 (9%)	n = 25 (7%)	n = 48 (17.0%)	n = 98 (27.5%)
1 Shock, n (%)*	84 (35.7%)	21 (32.8%)	12 (48.0%)	20 (41.7%)	31 (31.6%)
2–3 Shocks, n (%)*	48 (20.4%)	4 (6%)	3 (12.0%)	10 (20.8%)	31 (31.6%)
> 3 Shocks, n (%)*	50 (21.3%)	15 (23.4%)	2 (8%)	7 (14.6%)	26 (26.5%)
1 unknown, n (%)*	50 (21.3%)	24 (37.5%)	8 (32.0%)	11 (22.9%)	10 (10.2%)
Mechanical CPR, n (%)	47 (3%)	–	–	–	47 (13.2%)

Percentages were rounded, wherefore they might not add up to 100%. *Percentage calculated from the total of defibrillations (n = 235). **Percentage calculated from the total cases where advanced airway management with SGA or ETI was performed (n = 1390). ***Percentage calculated from the total number of the administered drug (N). *BMV* bag-mask ventilation; *SGA* supraglottic airway device; *SD* standard deviation; *CPR* cardiopulmonary resuscitation

### Outcome

Of 1,740 cases, the main proportion achieved no pre-hospital ROSC across all age groups.

The incidence of children declared dead on scene after unsuccessful resuscitation was 1.20/100,000, like those admitted to hospital with ROSC (1.13/100,000). In 46.9% (n = 343) in the group < 1 year, children were declared dead on scene, compared to 28.2% (n = 104) in 1 to 4 years, 31.8% (n = 90) in 5 to 12 years and 35.0% (n = 125) in 13 to 17 years. The incidences of hospital admittance were highest in 2019 (1.79/100,000) and 2015 (1.72/100,000).

Immediate- and short-term outcomes for all age groups are shown in Table 3. In total, 994 children did not achieve ROSC pre-hospital, with the majority being unwitnessed arrests and presenting with asystole as initial rhythm (Additional file 1: Table S6). Long-term outcome, where available, is presented in Additional file 1: Table S7. EMS time intervals did not differ between any short-term outcomes. An overall median response time of seven minutes (IQR 5 to 9) was observed. Univariate analysis showed no difference for any ROSC when EMS response time was below 7 min compared to above 7 min (OR 0.86; 95% CI 0.70 to 1.04) with a similar rate of bystander CPR (62.4% vs. 62.3%; p = 1.0). Across different outcomes, EMS time intervals did not differ (Additional file 1: Table S8).

**Table 3:** Short-term outcomes

	Overall N = 1,740	0– < 1 Year n = 731	1–4 Years n = 369	5–12 Years n = 283	13–17 Years n = 357
Status at hospital admission, n (%)/Incidence					
No admission, dead on scene	662 (38.0%)/1.20	343 (46.9%)	104 (28.2%)	90 (31.8%)	125 (35.0%)
Admitted with ongoing CPR	427 (24.5%)/0.75	170 (23.3%)	108 (29.3%)	74 (26.1%)	75 (21.0%)
Admitted with ROSC	650 (37.4%)/1.13	218 (29.8%)	157 (42.5%)	118 (41.7%)	157 (44.0%)
Unknown	1 (0.1%)	-	-	1 (0.4%)	-

*CPR* cardiopulmonary resuscitation, *ROSC* return of spontaneous circulation

### Bystander influencing immediate outcome

Chest compressions and ventilation started by bystanders had an adjusted odds ratio (aOR) for hospital admission with ROSC of 2.78 (95% CI 2.12–3.65), compared to CCV started by EMS. When only chest compressions were started by bystanders, aOR was also significant, with 1.72 (95% CI 1.38–2.14). OHCA witnessed by bystanders or EMS had increased aOR for hospital admission with ROSC compared to an unwitnessed OHCA. In addition to witness status and bystander CPR, the time of OHCA was analysed. Odds ratio was decreased during night-time for admission with ROSC (Fig. 3).

## Discussion

This paper is, to our knowledge, the first to give an epidemiological overview of OHCA in children, including patients in Germany between 2007 and 2021, and analyse modifiable factors for the occurrence of sustained ROSC at hospital admission.

Herein, 1,740 children were included, with the highest incidence of OHCA in the < 1 year group with 23.42/100,000. The majority of OHCA occurred at home in the youngest age group, with a change of OHCA to public places in older children. Trauma and drowning constituted a significant proportion of presumed causes in children above 1 year, while cardiac causes accounted for 17.6% in the oldest age group. The highest ROSC rate was observed in adolescents (44.0%; 157/357). As factors that can be modified through public awareness and training, bystander CCV and CCO had significantly higher aOR for hospital admission with ROSC. Confidence intervals of bystander CCO and CCV for hospital admission with ROSC barely overlap, indicating a possible significant difference in favour of CCV [25]. The importance of bystander ventilation and chest compression was found by a previous study, indicating that the results are likely to apply to both children and adults [26].

This points out the relevance of bystander-initiated ventilation in children suffering from OHCA even if survival to hospital discharge has not yet improved [27, 28].

One confounder might be that bystanders who perform CCV are better trained and provide higher-quality CPR than those who provide chest compressions ‘only’. The effect of CCV on (hospital admission with) ROSC has also been identified in adult studies and should be translated into future first aid courses and can also be implemented in childbirth preparation courses [26]. Although the group of first responders was small, it is important to differentiate between bystanders and first responders [29]. Bystanders will perform CPR by chance, whereas first responders perform CPR by system. The discussion of advanced airway management (AAM) during OHCA is ongoing, and multiple studies have focused on possible survival benefits. AAM had lower rates of good neurologic outcome in the French National OHCA Registry (4.6% vs. 11.1, in favour of the bag-mask ventilation (BMV) group) and Pan Asian Resuscitation Outcomes Study (1.8% vs. 6.8%, in favour of the BMV group) [2, 30]. These findings can be prone to selection bias, the airway paradox, and be influenced by the EMS system itself. On the contrary a significant higher aOR for AAM was found in this study. Due to the low incidence, recruitment of a significant sample size is associated with a very high effort, needing nationwide participation in a prospective trial. Therefore, prospective studies on AAM in children would be challenging to carry out.

Data on immediate outcome are presented herein as the rate of ROSC at hospital admission. This outcome has previously been reported in the range of 8.1% [5] to 38.7% [31] in various systems. As awareness for recognition of cardiac arrest has increased over the last couple of years, different study periods might partially explain these differences.

Age groups have not been uniformly defined across recent studies; however, children aged < 1 year consistently have the highest proportion of OHCA [32]. Longitudinal data show a trend towards a higher incidence of OHCA in children. This can be due to reporting bias, as GRR is a voluntary registry [21]. Across the study period, paediatric life support (PLS) guidelines have been updated several times. While in 2005, the PLS guideline stated that a capnometer ‘may be used’, a change in the 2010 PLS guidelines was observed as ‘capnography must be used’ [33, 34]. This change in guidelines and the ubiquitous availability of capnography is reflected in the increased rates of etCO2 at hospital admission. Necessity of rescue breaths has been stated in the 2015 PLS guidelines [35]. This is corroborated by our findings of a higher OR for hospital admission with ROSC. Although five initial rescue breaths are recommended if the person is trained to do so, this was not possible to determine due to the available data.

Based on this study, future EMS training should focus on children < 1 year in general and on the other age groups on treating trauma, drowning and hypoxia as reversible causes. In adults, when no obvious cause of OHCA is present, ‘cardiac’ is stated as the presumed cause; in children, a uniformly presumed cause in the absence of any clear reason for OHCA is undefined. This is mirrored in a combined share of 20.5% ‘unknown’ and ‘other causes’ within this study which corroborates a 30.0% of unknown causes in a recent study by Holgersen et al. [31]. Although it may not be possible to influence the timing of OHCA, the decreased aOR for night-time indicates there is a continuous need to evaluate monitoring devices, such as surveillance mattresses, for a potential role in aiding the early detection of cardiac or respiratory deterioration, alerting caregivers so as to prevent or recognise cardiac arrests immediately [36]. To our knowledge, there has not been a study showing the benefit of such devices; however, cardiac arrest registries could record the presence of any potentially preventive device. This must, however, be balanced against parent stress levels.

The high number (> 80%) of unknown outcomes at 24 h, 30 days and discharge suggests mandatory participation for hospitals receiving children with OHCA is necessary. Other registries were able to determine reliable 30-day outcomes [31, 37], enabled by linkage of different registries through a personal identification number. Data protection regulations should emphasise the use of anonymised or pseudonymised data to gain a better understanding of scarce patient populations.

Although this study presents previously scarcely reported data from one of the largest cardiac arrest registries in Europe, there are some limitations. (1) The latest Utstein recommendation on reporting OHCA in the paediatric population from 1995 proposes a reporting flowchart [11]. However, as current registry variables do not support the proposed format, such a chart was not applied in our study. (2) Due to the retrospective nature of this study, it was not possible to assess the rationale for different interventions like airway management [2, 30] or adrenaline dosage [13]. (3) The lack of long-term outcome data limits the significance of hospital admission with ROSC. (4) Due to the anonymised nature of data, it was impossible to contact the participating sites to clarify discrepancies or collect missing data. Missing data poses a challenge for any statistical analysis. Leading to a balancing act between feasibility for EMS providers and data completeness [38]. Where possible, missing data is presented in Additional file 1: Table S9. (5) Any information on bystanders is missing. Therefore, it is impossible to differentiate bystanders with and without medical backgrounds. (6) Determining the cause of death and providing this information to EMS providers can improve patient care. Even when a coroner evaluates the cause of death, 11.2% (49/474) remain undetermined, displaying high complexity and uncertainty [39].

Future studies should attempt to establish global collaborations to provide a complete picture of OHCA in the paediatric population. Existing registries should try to collect prospective information on treatment decisions to improve understanding of the rationale behind those decisions in this rare event.

## Conclusion

This study reports the incidence of OHCA in the paediatric population over a 15-year period in Germany. Chest compressions and ventilation by bystander are identified to have a higher impact on immediate survival compared with chest compression only. This study corroborates the findings of an increased incidence in < 1-year-olds and an overall low survival rate.

### Supplementary Information

**Additional file 1.** Supplementary tables.

## Data Availability

An aggregated dataset used during the current study is available from the corresponding author on reasonable request.

## Abbreviations

AAM: Advanced airway management
ALS: Advanced life support
aOR: Adjusted odds ratio
ASA: American Society of Anaesthesiologists Classification
CCO: Chest compression only
CCV: Chest compression and ventilation
CPC: Cerebral performance category
CPR: Cardiopulmonary resuscitation
DoA: Deceased on arrival
EMS: Emergency medical service
EMT: Emergency medical technician
ETI: Endotracheal intubation
EuReCa: European Registry of Cardiac Arrest
GRR: German Resuscitation Registry
i.o.: Intraosseous access
i.v.: Intravenous access
LT: Laryngeal tube
OHCA: Out-of-hospital cardiac arrest
PALS: Paediatric advanced life support
PEA: Pulseless electrical activity
RACA: Return of spontaneous circulation after cardiac arrest
ROSC: Return of spontaneous circulation
SD: Standard deviation
SIDS: Sudden infant death syndrome
STROBE: Strengthening the Reporting of Observational studies in Epidemiology
uaOR: Unadjusted odds ratio
VF: Ventricular fibrillation
VT: Ventricular tachycardia
