# Supplementary material for: Out-of-hospital cardiac arrest in children: an epidemiological study based on the German Resuscitation Registry identifying modifiable factors for return of spontaneous circulation
Source: Crit Care. 2023 Sep 7;27:349. doi: 10.1186/s13054-023-04630-3 (PMC10485980; doi:10.1186/s13054-023-04630-3)
Supplement: Supplementary file 1 — Additional file 1. Supplementary tables. [file 13054_2023_4630_MOESM1_ESM.docx]

**Supplemental Material to**

**Out-of-hospital cardiac arrest in children: An epidemiological study based on the German Resuscitation Registry identifying influencing factors for return of spontaneous circulation over a 15-year time span**

Stephan Katzenschlager^1*^, Inga K Kelpanides^2,3^, Patrick Ristau^4^, Matthias Huck^1^, Stephan Seewald^4,5^, Sebastian Brenner^6^, Florian Hoffmann^7^, Jan Wnent^4,5,8^, Jo Kramer-Johansen^3,4,9^, Ingvild B.M. Tjelmeland^3,4,9^, Markus A. Weigand^1^, Jan-Thorsten Gräsner^4,5^, Erik Popp^1^

1 Department of Anesthesiology, Heidelberg University Hospital, Heidelberg, Germany

2 Department of Research and Development, Division of Emergencies and Critical Care, Oslo University Hospital, Norway

3 Faculty of Medicine, Institute of Clinical Medicine, University of Oslo, Oslo, Norway

4 Institute for Emergency Medicine, University Hospital Schleswig-Holstein, Kiel, Germany

5 Department of Anesthesiology and Intensive Care Medicine, University Hospital Schleswig-Holstein, Campus Kiel, Kiel, Germany

6 Department of Pediatric and Adolescent Medicine, University Clinic Carl Gustav Carus, Dresden, Germany

7 Paediatric Intensive Care and Emergency Medicine, Dr. von Hauner Children's Hospital, Ludwig-Maximilians-University, Munich, Germany

8 School of Medicine, University of Namibia, Windhoek, Namibia

9 Division of Prehospital Services, Oslo University Hospital, Norway

* correspondence to:

Dr. med. univ. Stephan Katzenschlager

Department of Anaesthesiology, Heidelberg University Hospital, Germany

Im Neuenheimer Feld 420, 69120 Heidelberg, Germany

E-mail: [stephan.katzenschlager@med.uni-heidelberg.de](mailto:maximilian.dietrich@med.uni-heidelberg.de)

Phone: +49 6221 56 39683

**Table of content**

[Table 1 – (a) Population data over the study period (b) Incidence of pediatric OHCA 4](#_Toc143606861)

[Table 2 – Baseline characteristics of children ≤7 days 6](#_Toc143606862)

[Table 3 – Baseline characteristics of children without resuscitation attempts 8](#_Toc143606863)

[Table 4 – (a) Intubation performed by (b) Supraglottic airway device used by (c) Type of SGA used 9](#_Toc143606864)

[Table 5 – Application of advanced life support medication 10](#_Toc143606865)

[Table 6 – Circumstances and immediate outcome of children without prehospital return of spontaneous circulation 11](#_Toc143606866)

[Table 7 – Long term outcome 12](#_Toc143606867)

[Table 8 – EMS time intervals in different groups 12](#_Toc143606868)

[Table 9 – Missing data 14](#_Toc143606869)

# Table 1 – (a) Population data over the study period (b) Incidence of pediatric OHCA

1. Population data over the study period

|  | **2007** | **2008** | **2009** | **2010** | **2011** | **2012** | **2013** | **2014** | **2015** | **2016** | **2017** | **2018** | **2019** | **2020** | **2021** |
| --- | --- | --- | --- | --- | --- | --- | --- | --- | --- | --- | --- | --- | --- | --- | --- |
| Population Germany | 82,217,837 | 82,002,356 | 81,802,257 | 81,751,602 | 80,327,900 | 80,523,746 | 80,767,463 | 81,197,537 | 82,175,684 | 82,521,653 | 82,792,351 | 83,019,213 | 83,166,711 | 83,155,031 | 83,237,124 |
| Population within GRR | 8,361,654 | 10,782,322 | 11,106,462 | 14,174,186 | 15,413,411 | 17,008,222 | 19,113,556 | 23,872,791 | 24,365,935 | 25,698,482 | 27,651,143 | 32,001,893 | 33,433,118 | 32,812,224 | 31,180,058 |
| % of whole population within registry | 10% | 13% | 14% | 17% | 19% | 21% | 24% | 29% | 30% | 31% | 33% | 39% | 40% | 39% | 37% |
|  |  |  |  |  |  |  |  |  |  |  |  |  |  |  |  |
| Paediatric population across Germany | 1,371,311 | 1,768,301 | 1,821,460 | 2,324,567 | 2,527,799 | 2,772,340 | 3,096,396 | 3,843,519 | 3,947,281 | 4,188,853 | 4,534,787 | 5,248,310 | 5,483,031 | 5,414,017 | 5,207,070 |
| Paediatric population Germany / 100,000 | 13.7 | 17.7 | 18.2 | 23.2 | 25.3 | 27.7 | 31.0 | 38.4 | 39.5 | 41.9 | 45.3 | 52.5 | 54.8 | 54.1 | 52.1 |
| Calculated population per age group across Germany |  |  |  |  |  |  |  |  |  |  |  |  |  |  |  |
| 0-1 | 685,495 | 683,350 | 665,151 | 678,233 | 658,332 | 674,411 | 683,070 | 716,419 | 744,721 | 788,299 | 785,074 | 783,978 | 774,870 | 769,380 | 791,254 |
| 1-4 | 2,783,549 | 2,761,822 | 2,744,457 | 2,730,887 | 2,728,254 | 2,717,831 | 2,726,422 | 2,769,992 | 2,868,825 | 2,968,147 | 3,061,704 | 3,142,419 | 3,186,506 | 3,199,758 | 3,184,079 |
| 5-12 | 6,201,621 | 6,125,604 | 6,030,239 | 5,909,392 | 5,779,817 | 5,720,599 | 5,674,456 | 5,665,091 | 5,756,292 | 5,795,939 | 5,830,863 | 5,877,036 | 5,957,679 | 6,023,312 | 6,116,654 |
| 13-<18 | 4,299,418 | 4,112,781 | 4,041,846 | 4,022,377 | 3,976,549 | 3,978,046 | 3,991,581 | 3,960,518 | 3,955,839 | 3,917,877 | 3,860,505 | 3,793,995 | 3,758,847 | 3,751,494 | 3,771,272 |
| Calculated population per age group within registry | **Population per age group across Germany * % of whole population within registry** | | | | | | | | | | | | | | |
| 0-1 | 69,716 | 89,852 | 90,309 | 117,593 | 126,322 | 142,449 | 161,648 | 210,633 | 220,817 | 245,488 | 262,200 | 302,205 | 311,499 | 303,590 | 296,398 |
| 1-4 | 283,090 | 363,146 | 372,621 | 473,484 | 523,501 | 574,060 | 645,206 | 814,402 | 850,636 | 924,326 | 1,022,554 | 1,211,326 | 1,280,979 | 1,262,596 | 1,192,734 |
| 5-12 | 630,712 | 805,443 | 818,738 | 1,024,577 | 1,109,038 | 1,208,305 | 1,342,856 | 1,665,587 | 1,706,800 | 1,804,942 | 1,947,402 | 2,265,455 | 2,394,994 | 2,376,744 | 2,291,257 |
| 13-<18 | 437,256 | 540,781 | 548,770 | 697,404 | 763,025 | 840,243 | 944,604 | 1,164,427 | 1,172,947 | 1,220,086 | 1,289,339 | 1,462,493 | 1,511,061 | 1,480,306 | 1,412,693 |
| Per age group within registry /100,000 |  |  |  |  |  |  |  |  |  |  |  |  |  |  |  |
| 0-1 | 0.69 | 0.89 | 0.90 | 1.17 | 1.26 | 1.42 | 1.61 | 2.10 | 2.20 | 2.45 | 2.62 | 3.02 | 3.11 | 3.03 | 2.96 |
| 1-4 | 2.83 | 3.63 | 3.72 | 4.73 | 5.23 | 5.74 | 6.45 | 8.14 | 8.50 | 9.24 | 10.22 | 12.11 | 12.80 | 12.62 | 11.92 |
| 5-12 | 6.30 | 8.05 | 8.18 | 10.24 | 11.09 | 12.08 | 13.42 | 16.65 | 17.06 | 18.04 | 19.47 | 22.65 | 23.94 | 23.76 | 22.91 |
| 13-<18 | 4.37 | 5.40 | 5.48 | 6.97 | 7.63 | 8.40 | 9.44 | 11.64 | 11.72 | 12.20 | 12.89 | 14.62 | 15.11 | 14.80 | 14.12 |

1. Incidence of paediatric OHCA

|  | **2007** | **2008** | **2009** | **2010** | **2011** | **2012** | **2013** | **2014** | **2015** | **2016** | **2017** | **2018** | **2019** | **2020** | **2021** |
| --- | --- | --- | --- | --- | --- | --- | --- | --- | --- | --- | --- | --- | --- | --- | --- |
| Paediatric OHCA, N | 38 | 51 | 51 | 39 | 61 | 73 | 83 | 93 | 168 | 167 | 176 | 166 | 227 | 169 | 175 |
| Paediatrich OHCA per age group, n |  |  |  |  |  |  |  |  |  |  |  |  |  |  |  |
| 0-1 | 11 | 18 | 22 | 16 | 30 | 25 | 39 | 32 | 76 | 84 | 75 | 67 | 99 | 66 | 71 |
| 1-4 | 11 | 9 | 8 | 10 | 7 | 13 | 20 | 21 | 41 | 33 | 38 | 35 | 50 | 36 | 37 |
| 5-12 | 4 | 10 | 8 | 8 | 13 | 20 | 11 | 15 | 23 | 24 | 32 | 27 | 40 | 24 | 24 |
| 13-<18 | 12 | 15 | 14 | 6 | 11 | 15 | 13 | 25 | 28 | 26 | 31 | 37 | 38 | 43 | 43 |
| Incidence Overall | **Paediatric OHCA / Paediatric population Germany / 100,000** | | | | | | | | | | | | | | |
|  | 2.77 | 2.88 | 2.80 | 1.68 | 2.41 | 2.63 | 2.68 | 2.42 | 4.26 | 3.99 | 3.88 | 3.16 | 4.14 | 3.12 | 3.36 |
| Incidence per age group |  |  |  |  |  |  |  |  |  |  |  |  |  |  |  |
| 0-1 | 15.78 | 20.03 | 24.36 | 13.61 | 23.75 | 17.55 | 24.13 | 15.19 | 34.42 | 34.22 | 28.60 | 22.17 | 31.78 | 21.74 | 23.95 |
| 1-4 | 3.89 | 2.48 | 2.15 | 2.11 | 1.34 | 2.26 | 3.10 | 2.58 | 4.82 | 3.57 | 3.72 | 2.89 | 3.90 | 2.85 | 3.10 |
| 5-12 | 0.63 | 1.24 | 0.98 | 0.78 | 1.17 | 1.66 | 0.82 | 0.90 | 1.35 | 1.33 | 1.64 | 1.19 | 1.67 | 1.01 | 1.05 |
| 13-<18 | 2.74 | 2.77 | 2.55 | 0.86 | 1.44 | 1.79 | 1.38 | 2.15 | 2.39 | 2.13 | 2.40 | 2.53 | 2.51 | 2.90 | 3.04 |
|  |  |  |  |  |  |  |  |  |  |  |  |  |  |  |  |
| etCO2 at admission, n | 4 | 5 | 9 | 1 | 9 | 15 | 17 | 21 | 47 | 40 | 51 | 51 | 68 | 53 | 34 |

Abbreviation: GRR = German Resuscitation Registry

# Table 2 – Baseline characteristics of children ≤7 days

|  | **Overall**  **N = 97** |
| --- | --- |
| **Sex, n female (%)** | **40 (41.2%)** |
| **Location of OHCA, n (%)**  Home  Public Place  Healthcare facility  Other  Unknown | **46 (47.4%)**  **8 (8.2%)**  **9 (9.3%)**  **4 (4.1%)**  **30 (30.9%)** |
| **Presumed cause, n (%)**  Cardiac  Trauma  Hypoxia  SIDS  Intoxication  Other  Unknown | **17 (17.5%)**  **6 (6.2%)**  **31 (31.9%)**  **3 (3.1%)**  **4 (4.1%)**  **16 (16.5%)**  **20 (20.6%)** |
| **Witnessed by, n (%)**  Bystander  First Responder  EMS | **28 (28.8%)**  **5 (5.1%)**  **15 (15.4%)** |
| **First rhythm assessed, n (%)**  Bradycardia  Ventricular fibrillation  Pulseless electric activity  Asystole  no CPR performed | **11 (11.3%)**  **8 (8.2%)**  **12 (12.4%)**  **35 (36.1%)**  **28 (28.9%)** |
| **Immediate outcome, n (%)**  any ROSC  no ROSC  no CPR | **46 (47.4%)**  **29 (29.9%)**  **22 (22.7%)** |
| **Status at hospital admission, n (%)**  No admission, dead on scene  admitted with ongoing CPR  admitted with ROSC | **55 (56.7%)**  **9 (9.3%)**  **33 (34.0%)** |

Abbreviation: OHCA = out-of-hospital cardiac arrest; SIDS = sudden infant death syndrome; EMS = emergency medical service; CPR = cardiopulmonary resuscitation; ROSC = return of spontaneous circulation

# Table 3 – Baseline characteristics of children without resuscitation attempts

|  | **Overall**  **N = 476** | **0 – <1 Year**  **n = 254** | **1 – 4 Years**  **n = 37** | **5 – 12 Years**  **n = 41** | **13 – <18 Years**  **n = 144** |
| --- | --- | --- | --- | --- | --- |
| **Sex, n female (%)** | **177 (37.2%)** | **98 (38.6%)** | **12 (32.4%)** | **18 (43.9%)** | **49 (34.0%)** |
| **Location of OHCA, n (%)**  Home  Public Place  Educational Institution / Workplace  Other  Unknown | 36 (7.6%)  17 (3.6%)  1 (0.2%)  2 (0.4%)  121 (25.4%) | 10 (3.9%)  5 (2.0%)  -  1 (0.4%)  82 (32.3%) | 8 (21.6%)  2 (5.4%)  -  -  2 (5.4%) | 4 (9.8%)  2 (4.9%)  -  -  12 (29.3%) | 14 (9.7%)  8 (5.6%)  1 (0.7%)  1 (0.7%)  25 (17.4%) |
| **Presumed cause, n (%)**  Cardiac  Trauma  Drowning  Hypoxia  SIDS  Metabolic  Sepsis  Other  Unknown | 63 (13.2%)  82 (17.2%)  5 (1.1%)  51 (10.7%)  33 (6.9%)  5 (1.1%)  3 (0.6%)  51 (10.7%)  173 (36.3%) | 46 (18.1%)  18 (7.1%)  -  15 (5.9%)  28 (11.0%)  -  2 (0.8%)  23 (9.1%)  116 (45.7%) | 4 (10.8%)  7 (18.9%)  -  4 (10.8%)  5* (13.5%)  1 (2.7%)  -  3 (8.1%)  13 35.1%) | 4 (9.8%)  10 (24.4%)  2 (4.9%)  8 (19.5%)  -  2 (4.9%)  -  2 (4.9%)  13 (31.7%) | 9 (6.3%)  47 (32.6%)  3 (2.1%)  24 (16.7%)  -  2 (1.4%)  1 (0.7%)  23 (16.0%)  31 (21.5%) |

*SIDS was only reported in children up to 2 years of age. Abbreviations: OHCA = out-of-hospital cardiac arrest; SIDS = sudden infant death syndrome;


# Table 4 – (a) Intubation performed by (b) Supraglottic airway device used by (c) Type of SGA used

1. Intubation performed by

|  | **Overall**  **N = 1,070** | **0 – <1 Year**  **n = 420** | **1 – 4 Years**  **n = 221** | **5 – 12 Years**  **n = 193** | **13 – <18 Years**  **n = 236** |
| --- | --- | --- | --- | --- | --- |
| Other, n (%) | 12 (1.1%) | 3 (0.7%) | 4 (1.8%) | 3 (1.6%) | 2 (0.8%) |
| EMS, n (%) | 43 (4.0%) | 10 (2.4%) | 9 (4.1%) | 9 (4.7%) | 15 (6.4%) |
| Emergency Physician, n (%) | 1015 (94.9%) | 407 (96.9%) | 208 (94.1%) | 181 (93.8%) | 219 (92.8%) |

Abbreviations: EMS = Emergency Medical Service

1. Supraglottic airway device used by

|  | **Overall**  **N = 320** | **0 – <1 Year**  **n = 150** | **1 – 4 Years**  **n = 59** | **5 – 12 Years**  **n = 34** | **13 – <18 Years**  **n = 77** |
| --- | --- | --- | --- | --- | --- |
| Other, n (%) | 9 (2.8%) | 2 (1.3%) | 2 (3.4%) | 1 (2.9%) | 4 (5.2%) |
| EMS, n (%) | 105 (32.8%) | 41 (27.3%) | 11 (18.6%) | 10 (29.4%) | 43 (55.8%) |
| Emergency Physician, n (%) | 206 (64.4%) | 107 (71.3%) | 46 (78.0%) | 23 (67.6%) | 30 (39.0%) |

Abbreviations: EMS = Emergency Medical Service

1. type of supraglottic airway device used

| **Type SGA, n (%)** | **Overall**  **N = 320** | **0 – <1 Year**  **n = 150** | **1 – 4 Years**  **n = 59** | **5 – 12 Years**  **n = 34** | **13 – <18 Years**  **n = 77** |
| --- | --- | --- | --- | --- | --- |
| Laryngeal mask | 80 (25.0%) | 48 (32.0%) | 19 (32.2%) | 7 (20.6%) | 6 (7.8%) |
| Combitube | 3 (0.9%) | - | - | 2 (5.9%) | 1 (1.3%) |
| Others | 44 (13.8%) | 23 (15.3%) | 8 (13.6%) | 3 (8.8%) | 10 (13.0%) |
| Laryngeal tube | 175 (54.7%) | 71 (47.3%) | 26 (44.1%) | 21 (61.8%) | 57 (74.0%) |
| i-gel | 9 (2.8%) | 5 (3.3%) | 2 (3.4%) | - | 2 (2.6%) |
| Unknown | 9 (2.8%) | 3 (2.0%) | 4 (6.8%) | 1 (2.9%) | 1 (1.3%) |

Abbreviations: SGA = Supraglottic airway device; EMS = Emergency Medical Service

# Table 5 – Application of advanced life support medication

|  | **Overall**  **N = 1,740** | **0 – <1 Year**  **n = 731** | **1 – 4 Years**  **n = 369** | **5 – 12 Years**  **n = 283** | **13 – <18 Years**  **n = 357** |
| --- | --- | --- | --- | --- | --- |
| Adrenaline, e.b.; n (%)* | 30 (1.9%) | 19 (2.8%) | 8 (2.2%) | - | 3 (1.1%) |
| Adrenaline, CVC; n (%)* | 8 (0.5%) | 2 (0.3%) | 2 (0.6%) | 1 (0.4%) | 3 (1.1%) |
| Atropine, e.b.; n (%)* | 4 (2.3%) | 1 (1.9%) | 2 (3.6%) | - | 1 (3.2%) |
| Atropine, CVC; n (%)* | 1 (0.6%) | - | - | - | 1 (3.2%) |

* Percentage calculated from total number of applications, N stated in Table 2. Abbreviations: e.b. = endobronchial; CVC = central venous catheter

# Table 6 – Circumstances and immediate outcome of children without prehospital return of spontaneous circulation

| **First rhythm assessed, n (%)** | **Overall**  **N = 971** | **0 – <1 Year**  **n = 464** | **1 – 4 Years**  **n = 182** | **5 – 12 Years**  **n = 146** | **13 – <18 Years**  **n = 179** |
| --- | --- | --- | --- | --- | --- |
| Bradycardia | 19 (2.0%) | 9 (1.9%) | 6 (3.3%) | 1 (0.7%) | 3 (1.7%) |
| Ventricular fibrillation | 43 (4.4%) | 11 (2.4%) | 2 (1.1%) | 12 (8.2%) | 18 (10.1%) |
| Pulseless electric activity | 97 (10.0%) | 40 (8.6%) | 19 10.4%) | 16 (11.0%) | 22 (12.3%) |
| Asystole | 811 (83.5%) | 404 (87.1%) | 155 (85.2%) | 117 (80.1%) | 135 (75.4%) |
| unknown | 1 (0.1%) | - | - | - | 1 |

| **Witnessed by, n (%)** | **Overall**  **N = 994** | **0 – <1 Year**  **n = 473** | **1 – 4 Years**  **n = 187** | **5 – 12 Years**  **n = 149** | **13 – <18 Years**  **n = 185** |
| --- | --- | --- | --- | --- | --- |
| unwitnessed | 680 (68.4%) | 362 (76.5%) | 113 (60.4%) | 93 (62.4%) | 112 (60.5%) |
| First Responder witnessed | 12 (1.2%) | 6 (1.3%) | 3 (1.6%) | 1 (0.7%) | 2 (1.1%) |
| EMS witnessed | 52 (5.2%) | 18 (3.8%) | 9 (4.8%) | 12 (8.1%) | 13 (7.0%) |
| Bystander witnessed | 250 (25.2%) | 87 (18.4%) | 62 (33.2%) | 43 (28.9%) | 58 (31.4%) |

| **Status at hospital admission, n (%)** | **Overall**  **N = 994** | **0 – <1 Year**  **n = 473** | **1 – 4 Years**  **n = 187** | **5 – 12 Years**  **n = 149** | **13 – <18 Years**  **n = 185** |
| --- | --- | --- | --- | --- | --- |
| no admission, dead on scene | 649 (65.3%) | 335 (70.8%) | 103 (55.1%) | 89 (59.7%) | 122 (65.9%) |
| Admitted with ongoing CPR | 341 (34.3%) | 138 (29.2%) | 83 (44.4%) | 60 (40.3%) | 60 (32.4%) |

Abbreviations: ROSC = return of spontaneous circulation; CPR = cardiopulmonary resuscitation

# Table 7 – Long term outcome

| **Admitted to hospital** | **Overall**  **N = 1077 (61.9%)** | **0 – <1 Year**  **n = 388 (53.1%)** | **1 – 4 Years**  **n = 265 (71.8%)** | **5 – 12 Years**  **n = 192 (67.8%)** | **13 – <18 Years**  **n = 232 (65.0%)** |
| --- | --- | --- | --- | --- | --- |
| **Status at 24 hours*** |  |  |  |  |  |
| Alive, n (%) | 111 (10.3%) | 37 (9.5%) | 29 (10.9%) | 15 (7.8%) | 30 (12.9%) |
| Dead, n (%) | 69 (6.4%) | 26 (6.7%) | 17 (6.4%) | 16 (8.3%) | 10 (4.3%) |
| Unknown, n (%) | 897 (83.3%) | 325 (83.8%) | 219 (82.6%) | 161 (83.9%) | 192 (82.8%) |
| **Status at 30 days*** |  |  |  |  |  |
| Alive, n (%) | 58 (5.4%) | 18 (4.6%) | 16 (6.0%) | 9 (4.7%) | 15 (6.5%) |
| Dead, n (%) | 92 (8.5%) | 35 (9.0%) | 21 (7.9%) | 19 (9.9%) | 17 (7.3%) |
| Unknown, n (%) | 928 (86.2%) | 335 (86.3%) | 228 (86.0%) | 164 (85.4%) | 200 (86.2%) |
| **CPC-Score at discharge*** |  |  |  |  |  |
| CPC 1-2, n (%) | 40 (3.7%) | 17 (4.4%) | 9 (3.4%) | 5 (2.6%) | 9 (3.9%) |
| CPC 3-5, n (%) | 124 (11.5%) | 40 (10.3%) | 28 (10.6%) | 25 (13.0%) | 27 (11.6%) |
| Unknown, n (%) | 913 (84.8%) | 331 (85.3%) | 228 (86.0%) | 162 (84.4%) | 196 (84.5%) |

* Percentages calculated based on the proportion of patients with status “Admitted to hospital” Abbreviations: CPC = cerebral performance category

# Table 8 – EMS time intervals in different groups

1. Any ROSC

|  | Response time | On scene time | Transport time | Treatment time |
| --- | --- | --- | --- | --- |
| Median (Q1 to Q3) [mm:ss]; n | 7:00 (5:00 to 9:00); 713 | 36:00 (25:00 to 49:00); 599 | 12:00 (8:00 to 20:00); 572 | 52:00 (36:00 to 66:45); 564 |

1. No ROSC

|  | Response time | On scene time | Transport time | Treatment time |
| --- | --- | --- | --- | --- |
| Median (Q1 to Q3) [mm:ss]; n | 7:00 (5:00 to 10:00); 950 | 33:00 (25:00 to 43:00); 308 | 11:00 (7:00 to 16:45); 288 | 46:30 (36:00 to 59:00); 282 |

1. No hospital admission

|  | Response time | On scene time |
| --- | --- | --- |
| Median (Q1 to Q3) [mm:ss]; n | 7:00 (5:00 to 10:00); 630 | 36:00 (25:00 to 47:00); 15 |

1. Hospital admission with ROSC

|  | Response time | On scene time | Transport time | Treatment time |
| --- | --- | --- | --- | --- |
| Median (Q1 to Q3) [mm:ss]; n | 7:00 (5:00 to 9:00); 623 | 35:30 (25:00 to 48:15); 530 | 13:00 (8:00 to 20:00); 503 | 51:00 (36:00 to 67:00); 496 |

1. Hospital admission under CPR

|  | Response time | On scene time | Transport time | Treatment time |
| --- | --- | --- | --- | --- |
| Median (Q1 to Q3) [mm:ss]; n | 7:00 (5:00 to 9:00); 409 | 35:00 (26:00 to 46:00); 361 | 11:00 (7:00 to 16:45); 356 | 49:00 (36:00 to 60:30); 349 |

1. Time to first defibrillation

|  | First defribrillation |
| --- | --- |
| Median (Q1 to Q3) [mm:ss]; n | 12:00 (8:00 to 19:00); 159 |

# Table 9 – Missing data

|  | **Overall**  **N = 1,740** | **<1 Year**  **n = 731** | **1 – 4 Years**  **n = 369** | **5 – 12 Years**  **n = 283** | **13 – 17 Years**  **n = 357** |
| --- | --- | --- | --- | --- | --- |
| **Sex, n** | 3 | 1 | 0 | 2 | 0 |
| **Chest compression started by, n** | 78 | 31 | 18 | 7 | 22 |
| **Ventilation started by, n** | 443 | 173 | 85 | 76 | 109 |
| **Presumed cause, n** | 241 | 111 | 39 | 31 | 60 |
| **Nighttime (10pm to 6am), n** | 51 | 19 | 13 | 10 | 9 |
| **Pre Emergency Status, n** | 558 | 239 | 114 | 85 | 120 |
| **Defibrillation, total**  missing, n | **n = 235**  50 | **n = 64**  24 | **n = 25**  8 | **n = 48**  11 | **n = 98**  10 |
| **Epinephrine application, n** | 216 | 90 | 44 | 31 | 51 |
| **Amiodarone application, n** | 173 | 63 | 43 | 22 | 45 |
| **Atropine application, n** | 180 | 68 | 43 | 23 | 46 |
| **Status at hospital admission, n** | 1 |  |  | 1 |  |
